# Effects of long-term organic fertilizer and straw on soil quality and crop yield in a rapeseed–maize rotation system

**DOI:** 10.1371/journal.pone.0322223

**Published:** 2025-04-29

**Authors:** Xiaoqin Tian, Tingting Yang, Zhuo Li, Yonghong Liu

**Affiliations:** 1 Crop Research Institute, Sichuan Academy of Agricultural Sciences, Chengdu, China; 2 Provincial Key Laboratory of Water-Saving Agriculture in Hill Areas of Southern China, Chengdu, China; 3 Environment-friendly Crop Germplasm Innovation and Genetic Improvement Key Laboratory of Sichuan Province, Chengdu, China; 4 Chengdu Agricultural Technology Center, Chengdu, China; 5 Sichuan Academy of Agriculture Sciences, Chengdu, China; Directorate of Rapeseed-Mustard Research, INDIA

## Abstract

This study aimed to pursue more sustainable agriculture in a new rapeseed–maize rotation system. We assessed the effects of organic fertilizer and straw on soil quality and crop yield through a 15-year field experiment: mainly applied organic fertilizer (MM); mainly applied inorganic fertilizer with straw (MCS); mainly applied inorganic fertilizer (MC); applied only inorganic fertilizer with straw (CS); and applied only inorganic fertilizer (C) as a control. Compared to the C treatment, crop yields, soil organic carbon (SOC), available nitrogen (AN), phosphorus (AP), potassium (AK), soil porosity (SP), field capacity (FC), and 0.250–2.000 mm large aggregates in soil water-stable aggregates (WA0.250-2.000) were significantly (*P*<0.05) increased by organic fertilization treatments (MCS, MC, and MM); and soil bulk density (SBD) and WA<0.053 were significantly decreased. Similarly, straw addition (CS and MCS) treatments also had significant effects on soil nutrients, structure, and yield, compared to the C and MC treatments, respectively. However, compared to the MC treatment, applying more organic fertilizers (MM) had no significant effect. The highest nutrient comprehensive evaluation value (NCEV), SP, FC, MA>2.000, WA0.053–0.250, and crop yields were observed in the MCS treatment. Compared to C treatment, rapeseed and maize yield significantly increased by 36.0% and 11.8% in response to MCS treatment, respectively. Pearson correlations showed that total nitrogen (TN), total potassium (TK), AK, SBD, and WA<0.053 were the strongest correlates of crop yields, followed by SOC, FC, WA>2, and WA0.250–2.000. This suggests that MCS was the best fertilization method to increase crop yields by improving SOC, AK, FC, and WA>0.250, and reducing SBD and WA<0.053.

## 1 Introduction

There are upland sloping fields of approximately 230 million acres in Southwest China, accounting for 62% of the cultivated area [1]. It is an advantageous area for summer maize and winter rapeseed and is one of the largest imported regions for rapeseed oil in China [2]. The summer maize and winter rapeseed rotation system is a new double cropping model and a main cultivation model in arid sloping fields in southwest China. It has developmental potential in agricultural production due to its simplified field management, suitability for large-scale production, and integration of primary and secondary industries [2]. Inorganic fertilization plays an important role in agricultural production and is commonly used to compensate for the loss of soil nutrients during crop growth stages to ensure the supply of grain and oil safety supply [3]. However, continuous and single inorganic fertilization have adverse effects on soil health and long-term productivity, such as decreased nutrient use efficiency, accelerated soil organic carbon loss, intensified soil acidification, and damaged soil structure [4]. Therefore, increasing crop yield and controlling the adverse effects of inorganic fertilization have become a research focus in sustainable agricultural development.

To alleviate the decline in soil quality caused by the extensive use of inorganic fertilization in farmland, the application of organic fertilization has been continuously developed. In recent years, the combination of organic and inorganic fertilization has become one of the most active and rapidly developing fields. The application of organic matter (straw and livestock manure) is an important soil management measure that not only provides nutrients for plants but also maintains soil fertility by supplementing organic matter. In addition to being used as grain and oil crops, the by-products of rapeseed and maize, straws, contain abundant nutrients, such as nitrogen, phosphorus, and potassium, as well as organic compounds, such as cellulose, hemicellulose, lignin, protein, and carbohydrates. After returning to the field for utilization, it can provide essential nutrients for the growth of subsequent crops and increase crop yields. Ayesha et al. [5] showed that applying vermicompost to soil lowered its pH, improved porosity and water retention capacity, and enhanced microbial activity and overall soil health. In addition, using vermicompost alone as a nutrient source not only boosted yields by 21.31% over conventional fertilizers but also enhanced the nutritional value and cooking quality [6]. However, the application of manure alone may result in a slow nutrient release rate and a long release period [7]. The combination of organic and inorganic fertilization has the advantages of quick and long-term effects, which can enhance soil quality and have a significant positive impact on grain and oil production [8].

Numerous studies have shown that the combined application of chemical fertilizers and straw or manure is an effective measure for maintaining soil health through rational utilization of organic nutrient resources [9,10]. It can not only partially replace chemical fertilizers and effectively reduce inorganic manure, but it can also improve soil fertility and crop yield by increasing soil nutrients, soil organic matter content, soil structure and water retention, and beneficial microorganisms [11]. Xie et al. [12] showed that long-term inorganic and organic fertilization increases soil organic matter and amorphous oxides. However, different proportions of organic and inorganic fertilization applications can also greatly affect soil fertility and crop yield. For example, Dai et al. [13] suggested that the substitution of 70% chemical N with organic N is the most effective fertilizer practice for improving soil fertility and rice yield in double rice cropping systems. Wu et al. [14] found that partial organic substitution for synthetic fertilizer, especially 45% organic substitution, would be a feasible fertilization strategy to improve soil fertility and crop productivity while mitigating N_2_O emissions in wheat-maize rotation systems. Similarly, Song et al. [15] showed that a lower proportion of organic fertilization application had the best effect on crop yield increase in double rice cropping systems. The effects of different proportions of organic and inorganic fertilization on soil nutrient status, soil structure, and crop yield vary greatly in different agricultural ecosystems, and there is a lack of research on rapeseed–maize rotation systems.

Therefore, the long-term field experiment was conducted to evaluate the influence of different proportions of organic fertilization (including straw) on soil nutrient status, soil structure and crop yield, and explore the main soil factors affecting the crop yield, leading to best fertilization management measures that ensure consistently high crop yield. Experimental results are expected to provide important theoretical basis and technical support in the rapeseed–maize rotation system in the southwestern arid areas of China.

## 2 Materials and methods

### 2.1 Field experiments

Field experiments were conducted at the Xindu Experimental Station of the Sichuan Academy of Agricultural Sciences in Chengdu, Sichuan Province (30^◦^83^′^ N, 104^◦^15^′^ E). The mean annual temperature and rainfall at this site were 16.1 ^◦^C and 885 mm, respectively. According to the classification of the World Reference Base (WRB) for Soil Resources [16], the soil at the location of the field experiment can be classified as calcaric epileptic regosols (Loamic, Ochric). The Chinese soil classification system uses purple soil.

The field experiment followed a rapeseed–maize rotation system and started testing multiple fertilization schemes in 2008. The soil of the experimental field was divided into 5.0 m × 20.0 m plots, each separated by 30-cm cement barriers. The soil chemical properties were: pH 7.80, organic matter 6.29 g kg^-1^, total nitrogen 0.65 g kg^-1^, total phosphorus 0.32 g kg^-1^, total potassium 9.93 g kg^-1^, available nitrogen 37.70 mg kg^-1^, available phosphorus 8.03 mg kg^-1^, available potassium 120.33 mg kg^-1^. Plots requiring standard inorganic nitrogen, phosphorus, and potassium dosage were treated with 650 kg ha^-1^ urea (46% N), 870 kg ha^-1^ calcium superphosphate (16% P_2_O_5_), and 330 kg ha^-1^ potassium chloride (60% K_2_O) in a rapeseed–maize rotation system. Plots were distributed in a completely randomized block design with three replications for each treatment for a total of 15 plots. The treatments were as follows: mainly applied organic fertilizer (MM); mainly applied inorganic fertilizer with straw (MCS); mainly applied inorganic fertilizer (MC); applied only inorganic fertilizer with straw (CS); and applied only inorganic fertilizer (C). Plots requiring commercial organic fertilizer (contained approximately 450 g kg^-1^ carbon, 40 g kg^-1^ available nitrogen, 30 g kg^-1^ available phosphorus, 28 g kg^-1^ available potassium, and 300 g kg^-1^ water content) were purchased from Sichuan Guoguang Agrochemical Co., Ltd. The fertilization rates for rapeseed and maize were 40% and 60% of the total fertilizer, respectively. Inorganic and organic fertilizers were applied basally during plowing. The treatments with straw were to evenly spread the straw of previous crop among the rows of current crops before current crop closure (Table 1).

**Table 1:** Annual input amount of N, P_2_O_5_ and K_2_O in organic and inorganic fertilizers at the experimental site.

Crop	Treatment	Organic fertilizer (kg ha^–1^)				Chemical fertilizer (kg ha^–1^)						Straw or not
		Organic fertilizer	N	P_2_O_5_	K_2_O	Urea	N	Calcium superphosphate	P_2_O_5_	Potassium chloride	K_2_O	
Maize	MM	4004	112	84	79	146	67	0	0	67	40	No
	MCS	2002	56	42	39	268	123	260	42	133	80	Yes
	MC	2002	56	42	39	268	123	260	42	133	80	No
	CS	0	0	0	0	390	179	522	84	198	119	Yes
	C	0	0	0	0	390	179	522	84	198	119	No
Rapeseed	MM	2669	75	56	52	97	45	0	0	45	27	No
	MCS	1334	38	28	26	179	82	173	28	88	53	Yes
	MC	1334	38	28	26	179	82	173	28	88	53	No
	CS	0	0	0	0	260	120	348	56	132	79	Yes
	C	0	0	0	0	260	120	348	56	132	79	No
Total	MM	6673	187	140	131	243	112	0	0	112	67	No
	MCS	3336	93	70	65	447	206	433	70	221	133	Yes
	MC	3336	93	70	65	447	206	433	70	221	133	No
	CS	0	0	0	0	650	299	870	140	330	198	Yes
	C	0	0	0	0	650	299	870	140	330	198	No

MM, mainly applied organic fertilizer; MCS, mainly applied inorganic fertilizer with straw; MC, mainly applied inorganic fertilizer; CS, applied only inorganic fertilizer with straw; C, applied only inorganic fertilizer.

Both rapeseed and maize were cultivated using the traditional methods. Rapeseed (variety Chuanyou 81) and maize (variety Chengdan 922) were used in the experiment, with planting densities of 180,000 and 52,500 plants ha^-1^, respectively. Maize was sown in June and harvested in September; rapeseed was sown in October and harvested in May of the following year. Weed, pesticide, and pest management practices were the same as those of local farmers.

### 2.2 Soil sampling and analyses

Topsoil samples (0–20 cm layer) were collected using an auger from the downslope, mid-slope, and up-slope in each plot after rapeseed harvest in May 2023 and brought back to the laboratory for analysis [17]. Soil analyses were performed on air-dried and 2-mm sieved samples. Soil physicochemical properties were determined following the methods of Tian (2021) [18]. Soil organic carbon (SOC) was measured using the potassium dichromate oxidation method. Total nitrogen (TN) and total phosphorus (TP) were determined by the Kjeldahl method and the digestion-Mo-Sb anti-spectrophotometric method, respectively. Alkaline nitrogen (AN) and available phosphorus (AP) were assessed using alkaline hydrolysis diffusion and molybdenum blue methods, respectively. Total potassium (TK) and available potassium (AK) were measured with a flame photometer using ammonium acetate (NH₄OAc) digestion.

The following equation was used to calculate nutrient comprehensive evaluation value (NCEV) [19].


$$R_{m}=A_{m}/A_{mc}$$

$$\text{NCEV }=\frac{1}{n}\sum_{m=1}^{n}R_{m}（n=1，2，\ldots 7）$$

where NCEV is the comprehensive evaluation value of seven indicators (SOC, TN, TP, TK, AN, AP, and AK), A_m_ is the measured value of the m-th indicator in a certain treatment, A_mc_ is the measured value of the m-th indicator in the C treatment, and Rm is the relative value of the m-th indicator in a certain treatment.

At the same time as sampling the topsoil, undisturbed soil samples were collected using a 100 cm^3^ (*V*) ring knife. The soil ring (*m*_*1*_) was then placed vertically downward with the perforated end into an iron tray (the depth of the tray being 8–10 cm), and distilled water was slowly added to the tray, causing it to rise slowly to the upper edge of the soil ring. The sample was soaked for 12 h, with additional distilled water added during the soaking process. Following the saturation process, the soil ring was carefully transferred, without inverting it, onto a flat tray filled with dry sand. Then, the soil ring was left in place for an additional 24 h. After this period, it was weighed (*m*_*2*_) to measure the field capacity (FC). After the above weighing (*m*_*2*_), take all the soil from the soil ring and placed it into a pre-weighed aluminum box (*m*_*3*_). Then placed in a drying oven at 105°C ± 2°C until a constant mass (*m*_*4*_) was achieved. The soil bulk density (SBD) and field capacity (FC) were calculated [20]. The calculation formula is:


$$\text{SBD}=\left(m_{4}-m_{3}\right)/V$$

$$\text{FC}=\left[\left(m_{2}-m_{1}\right)-\left(m_{4}-m_{3}\right)\right]/\left(m_{4}-m_{3}\right)$$

The soil porosity (SP) is calculated using the formula [20]:


SP=100-SBD/2.65*100

where 2.65 is the soil’s specific gravity.

In addition, 1 kg of undisturbed soil was collected using a shovel with gravel, plant roots, debris, and other impurities removed, and then broken into small pieces along the soil’s natural structure to measure the soil mechanical-stable aggregates (MA) and water-stable aggregates (WA) using dry and wet sieving methods, respectively. The dry sieving method [18] was employed to analyze the undisturbed air-dried soil. The soil was placed on a sieve mesh, which was arranged in order from top to bottom according to the size of the sieve holes (>2, 0.25–2, 0.053–0.25, <0.053 mm). The soil was then placed on a shaking table at a speed of 60 times/minute and shaken for 2 min. Subsequently, soil samples were collected and weighed from each sieve mesh level in order to calculate the percentage content of soil aggregates at each particle level. The wet sieve method [21] was employed to ascertain the water stability of soil aggregates, with the percentage content of each particle size obtained by the dry sieve method serving as the basis for the determination. A total of 50 g of mixed soil samples were combined in accordance with the appropriate ratio. The sieve mesh was arranged in order from large to small and placed on the oscillating frame of the wet sieve instrument, ensuring that the upper and lower oscillations were fully immersed in the water. The instrument was operated at a frequency of 30 times/minute, with an amplitude of 3 cm, for a period of 30 min. Once the wet sieving process was complete, the soil particles of each size were gently rinsed into the evaporation dish using deionised water. After evaporation, the soil aggregates of each particle size were weighed and calculated.

### 2.3 Rapeseed and maize analyses

At crops physiological maturity, all plants in each plot were separately harvested to evaluate the population yield (grain yield per unit area) after sun drying.

### 2.4 Statistical analysis

The response of each indicator to different fertilization treatmens was analyzed using one-way analysis of variance (ANOVA). Differences among all treatments were detected using S-N-K test at a significance level of 0.05. The data were expressed as mean ± standard deviation values (*x̅*±s). This analysis was conducted using SPSS (version 17.0, SPSS Inc., Chicago, IL, USA). To visualize differences in crop yield, which may be explained by soil nutrient and structural variables, Pearson correlations and Mantel tests were visualized using R (v4.1.1) software via the “linkET” package.

## 3 Results

### 3.1 Plant nutrients in soils

Long-term fertilization measures had a significant impact on SOC, AN, AP, and AK (Table 2), but had no significant (*P*>0.05) effects on TN, TP, and TK. SOC, AN and AK were significantly higher among plots treated with organic fertilization (MCS, MC, and MM) than in the CS and C treatments. However, applying more organic fertilizers (MM) only significantly increase SOC, compared to the MC treatment. Similarly, straw addition also significantly improved soil nutrients, and the improvement effect varied depending on whether organic fertilizer was added or not. Compared to the C treatment, CS treatment significantly improved SOC, and MCS treatment significantly increased AN and AP than the MC treatment. The nutrient comprehensive evaluation value (NCEV) of MCS treatment was the highest.

**Table 2:** Effects of long-term organic fertilizer and straw on soil nutrients in a rapeseed–maize rotation system.

Treatment	SOC (g kg^-1^)	TN (g kg^-1^)	TP (g kg^-1^)	TK (g kg^-1^)	AN (mg kg^-1^)	AP (mg kg^-1^)	AK (mg kg^-1^)	NCEV
MM	10.57±0.05a	0.78±0.03a	0.38±0.06a	10.13±0.72a	54.07±3.68b	11.50±0.89b	156.00±5.00a	1.23
MCS	9.66±0.08b	0.82±0.04a	0.50±0.08a	10.27±0.21a	66.02±5.71a	14.56±0.91a	164.33±10.21a	1.37
MC	9.65±0.33b	0.80±0.12a	0.43±0.10a	10.23±0.29a	54.13±5.05b	11.53±0.70b	157.33±4.93a	1.24
CS	8.82±0.28c	0.74±0.02a	0.34±0.13a	10.10±0.61a	45.13±3.17c	10.17±0.59bc	137.00±6.56b	1.10
C	6.62±0.23d	0.70±0.06a	0.33±0.01a	9.93±0.51a	41.66±1.92c	9.60±0.89c	127.33±5.51bc	1.00

*Values are presented as the mean ± standard error from three biological replicate plots. Different lower case letters indicate significant differences according to the S-N-K test at *P* ≤ 0.05. SOC, soil organic carbon; TN, total nitrogen; TP, total phosphorus; TK, total potassium; AN, alkali hydrolyzed nitrogen; AP, available phosphorus; AK, available potassium; NCEV, nutrient comprehensive evaluation value; MM, mainly applied organic fertilizer; MCS, mainly applied inorganic fertilizer with straw; MC, mainly applied inorganic fertilizer; CS, applied only inorganic fertilizer with straw; C, applied only inorganic fertilizer.

### 3.2 Soil bulk density, porosity, filed capacity

**Fig 1:**
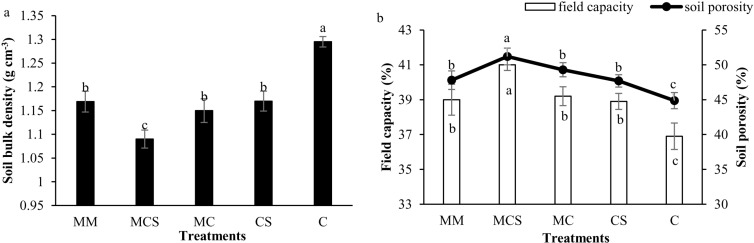
Effects of long-term organic fertilizer and straw on soil bulk density (a), soil porosity (b) and field capacity (b) in a rapeseed–maize rotation system. Different lower case letters indicate significant differences according to the S-N-K test at *P* ≤ 0.05. MM, mainly applied organic fertilizer; MCS, mainly applied inorganic fertilizer with straw; MC, mainly applied inorganic fertilizer; CS, applied only inorganic fertilizer with straw; C, applied only inorganic fertilizer.

Long-term fertilization regimes also had significant effects on soil bulk density, porosity, and filed capacity (Figs 1a and 1b). Specifically, long-term organic fertilization (MCS, MC, and MM) and straw addition (CS) treatments significantly increased soil porosity and filed capacity, compared with the C treatments, also significantly decreased soil bulk density. Besides, compared to the MC treatment, MCS treatment also significantly improved soil porosity and filed capacity, and decreased soil bulk density. Nonetheless, applying more organic fertilizers (MM) had no significant differences compared to the MC treatment. The highest soil porosity and filed capacity, and the lowest soil bulk density were observed in the MCS treatment (significant differences compared to other treatments).

### 3.3 Soil aggregates

**Fig 2:**
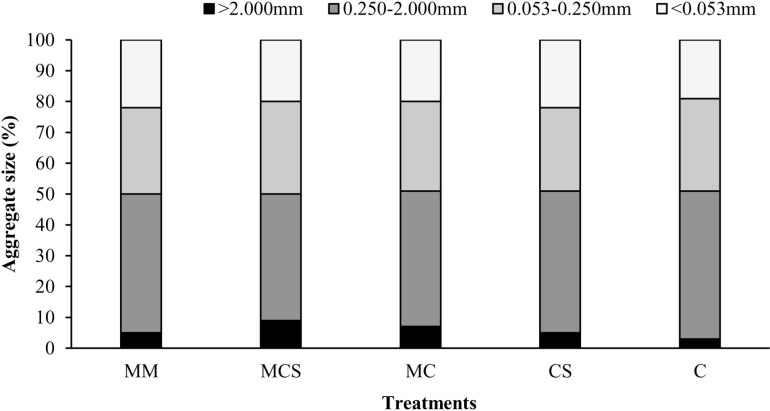
Effects of long-term organic fertilizer and straw on content of soil mechanical-stable aggregates in a rapeseed–maize rotation system. MM, mainly applied organic fertilizer; MCS, mainly applied inorganic fertilizer with straw; MC, mainly applied inorganic fertilizer; CS, applied only inorganic fertilizer with straw; C, applied only inorganic fertilizer.

As shown in Fig 2, after classifying the soil aggregates (0–20 cm) in each treatment, the dominant particle sizes were 0.250–2.000 mm and 0.053–0.250 mm aggregates, which accounted for 45.0% and 29.7% of the total soil aggregates, respectively, which were significantly higher than the other two particle sizes. The proportion of >2.000 mm larger aggregates was the smallest. Compared with C treatments, long-term organic fertilization (MCS, MC, and MM) and straw addition (CS) treatments promoted the content of >2.000 mm larger aggregates and correspondingly reduced the proportion of 0.250–2.000 mm large aggregates, while having no significant difference.

The soil water-stable aggregates under different long-term fertilization regimes are reported in Table 3, which shows different trends. Overall, the content of the 0.250–2.000 mm aggregates was the highest, followed by the 0.053–0.250 mm aggregates, and the other two particle sizes were relatively low. Long-term organic fertilization (MCS, MC, and MM) and straw addition (CS) treatments significantly increased the content of 0.250–2.000 mm large aggregates compared with the C treatment, as it significantly reduced the content of <0.053 mm aggregates. The content of 0.053–0.250 mm micro aggregates was significantly higher in the MCS group than in all other treatments, and the other treatments showed no significant difference compared to C treatment. The proportion of >2.000 mm larger aggregates treated with organic fertilization (except for MM) was significantly higher than that in the C treatment. Compared to the MC treatment, applying more organic fertilizers (MM) had no significant differences in various particle size aggregates.

**Table 3:** Effects of long-term organic fertilizer and straw on content of soil water-stable aggregates in a rapeseed–maize rotation system.

Treatments	Aggregate size (%)			
	>2.000 mm	0.250-2.000 mm	0.053-0.250 mm	<0.053 mm
MM	3.9±0.1ab	48.1±1.1a	35.9±0.2b	12.1±0.6b
MCS	4.0±0.1a	48.0±1.2a	36.7±0.1a	11.3±0.4b
MC	4.0±0.1a	48.3±1.0a	36.0±0.2b	11.7±0.4b
CS	3.9±0.1ab	48.1±1.1a	35.8±0.3b	12.2±0.3b
C	3.8±0.0b	45.2±1.4b	36.1±0.1b	14.9±0.3a

*Values are presented as the mean ± standard error from three biological replicate plots. Different lower case letters indicate significant differences according to the S-N-K test at *P* ≤ 0.05. MM, mainly applied organic fertilizer; MCS, mainly applied inorganic fertilizer with straw; MC, mainly applied inorganic fertilizer; CS, applied only inorganic fertilizer with straw; C, applied only inorganic fertilizer.

### 3.4 Rapeseed and maize yields

**Fig 3:**
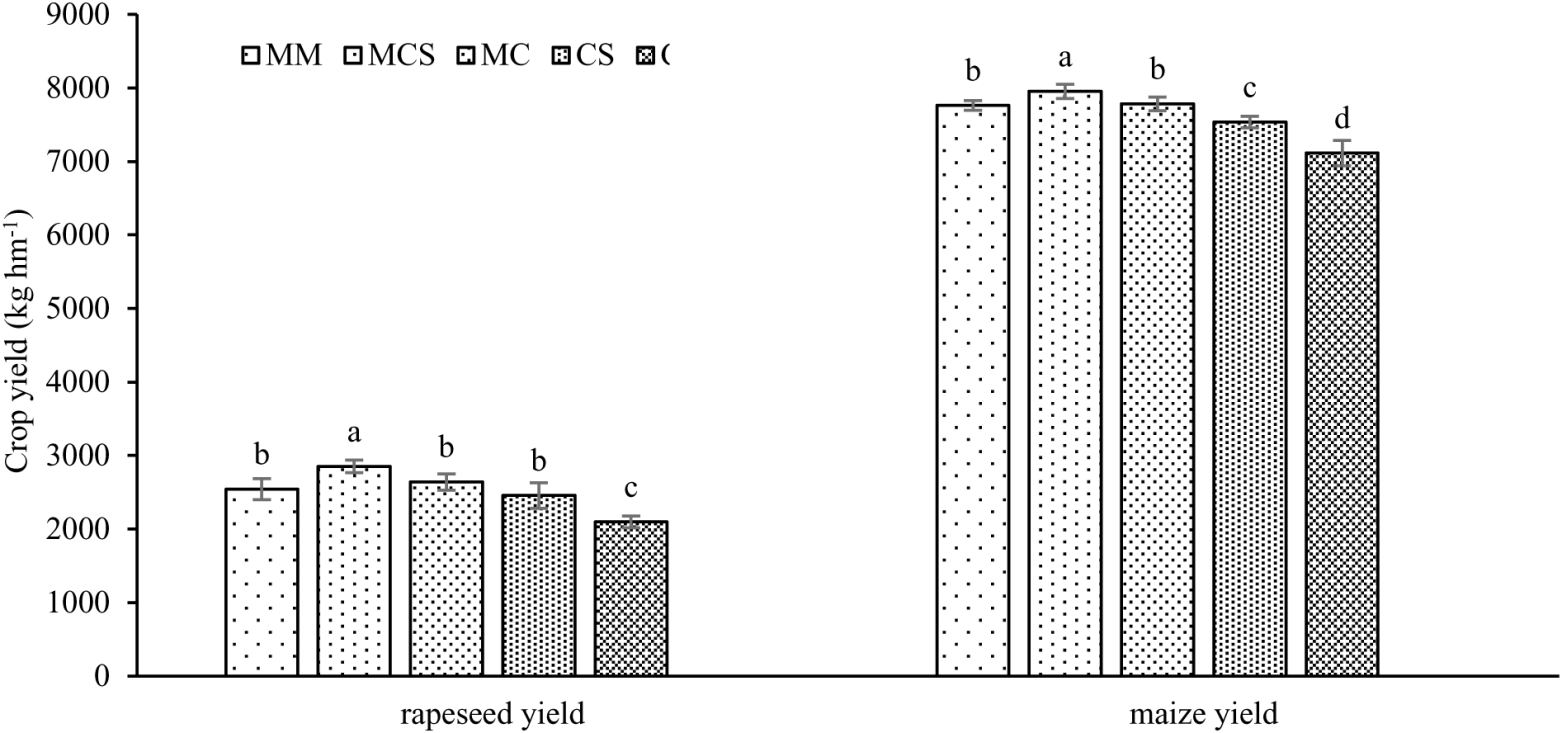
Effects of long-term organic fertilizer and straw on rapeseed and maize yield. Different lower case letters indicate significant differences according to the S-N-K test at *P* ≤ 0.05. MM, mainly applied organic fertilizer; MCS, mainly applied inorganic fertilizer with straw; MC, mainly applied inorganic fertilizer; CS, applied only inorganic fertilizer with straw; C, applied only inorganic fertilizer.

Crop yield were significantly impacted by long-term organic fertilizer and straw (Fig 3). In the rapeseed–maize rotation system, the yield of MCS treatment was the highest, followed by MC, MM, CS, and C treatments. Compared to C treatment, rapeseed yield significantly increased by 36.0%, 25.8%, 21.2%, and 17.0%; maize yield significantly increased by 11.8%, 9.4%, 9.1%, and 5.9% in response to MCS, MC, MM, and CS treatments, respectively. Besides, compared to the MC treatment, MCS treatment also significantly increased rapeseed and maize yield. Whereas, applying more organic fertilizers (MM) had no significant differences and MC treatment in rapeseed and maize yield.

### 3.5 Relationship between crop yields and soil properties

**Fig 4:**
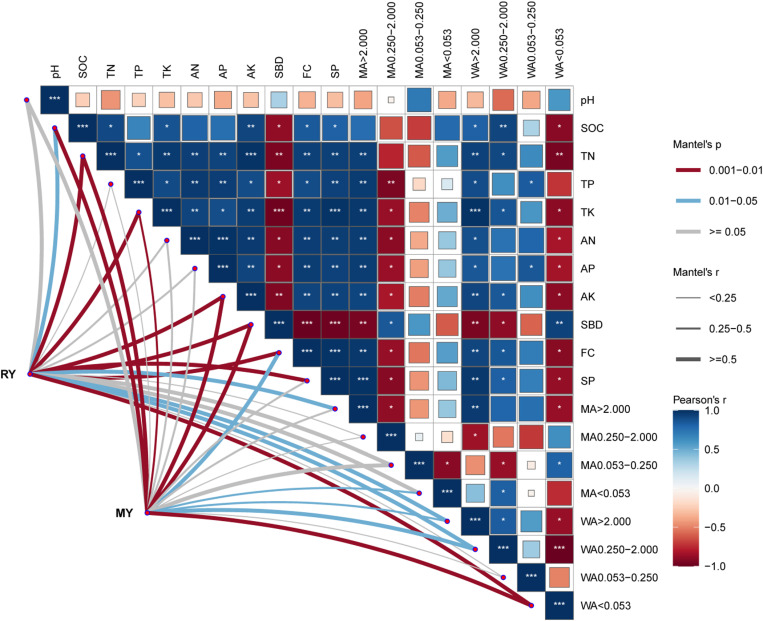
Soil environmental drivers of crops yield. *Pairwise comparisons of environmental variables were calculated by use of Pearson correlations. RY and MY were related to each environmental variable by Mantel tests. Edge width corresponds to the Mantel’s *r* statistic for the corresponding distance correlations, and edge color denoted the statistical significance based on 9,999 permutations. RY, rapeseed yield; MY, maize yield; SOC, soil organic carbon; TN, total nitrogen; TP, total phosphorus; TK, total potassium; AN, alkali hydrolyzed nitrogen; AP, available phosphorus; AK, available potassium; SBD, soil bulk density; FC, field capacity; SP, soil porosity; MA, mechanical-stable aggregates and WA, water-stable aggregates.

To identify the soil environmental drivers of crop yield, we correlated the distance-corrected dissimilarities of rapeseed and maize yield with those of environmental factors (Fig 4). Overall, TN, TK, AK, SBD, and WA<0.053 were the strongest correlates of both rapeseed and maize yields, followed by SOC, FC, WA>2, and WA0.250–2.000, whereas no significant correlations were found for pH, TP, AN, AP, MA0.250–2.000, MA0.053–0.250, and WA0.053–0.250. In addition, SP and MA>2 were only correlated with rapeseed yield, and MA<0.053 was only correlated with maize yield.

## 4 Discussion

### 4.1 Impacts of organic fertilization and straw on soil properties

In this study, organic fertilization (MCS, MC, and MM) treatments effectively boosted SOC, AN, AP, and AK contents compared to other fertilization treatments (Table 2). Organic amendments themselves contain abundant organic carbon (contained approximately 450 g kg^-1^ carbon), which could also create favorable conditions for microbial activity because of balanced sources of C and N substrates [22], improving crop root and shoot growth, and leading to a subsequent increase in SOC accumulation [23]. In addition, the combination of organic and inorganic fertilization could slow down the release and loss of nutrients as well as improve nutrient utilization efficiency [24,25], resulting in an increase in available nutrients such as AN, AP, and AK. Hu et al. [26] also found that the application of organic and inorganic fertilizers increased soil phosphatase activity, promoted soil phosphorus cycling, and significantly increased the AP content. Similarly, straw addition also significantly improved SOC, AN and AP (Table 2). During the degradation process of straw, a large amount of organic carbon and nitrogen substances were transported to the soil to promote the growth and reproduction of microorganisms. Besides, straw also produced organic matter such as polysaccharides, proteins, lignin, and nitrogen, phosphorus, potassium elements, thereby increasing the soil nutrient content [27].

Long-term application of inorganic fertilization could lead to soil compaction, while the combination of organic and inorganic fertilization effectively alleviated the trend of compaction [28]. Consistent with the above research results, compared with the C treatment, long-term organic fertilization (MCS, MC, and MM) and straw addition (CS) treatments significantly increased SP and FC, and decreased SBD. This was likely due to the increase in soil organic C (Table 2), which improved the soil structure, thus increasing SP and FC, and decreasing SBD [29]. Liu et al. [30] also indicated that the combination of inorganic and organic fertilizers (including straw) could effectively improve the soil environment, resulting in a loose soil texture and increased soil permeability.

Compared to chemical fertilizer (C), long-term organic fertilization (MCS, MC, and MM) and straw addition (CS) treatments both significantly increased WA0.250–2.000. The primary reason may be that organic fertilization and straw addition increase soil carbon input (Table 2), providing more organic residues for the soil and promoting the formation of large aggregates [31]. Another reason could be that organic matter is an important source of fulvic acid in soil aggregates, which could provide a good living environment for soil microorganisms and enhance microbial activity, thus promoting the cementation of small-particle aggregates and the formation of large-particle aggregates [32].

Besides, in this study, compared to the MC treatment, applying more organic fertilizers (MM) only significantly increased the organic carbon, but had no significant effect on soil nutrients, structure, and aggregates, as the amount of chemical fertilizers correspondingly decreased.

### 4.2 Effects of organic fertilization and straw on crop yields

In this study, MCS, MC and MM treatments achieved higher yields (rapeseed and maize), compared to C and CS treatmnets. These results are consistent with previous studies showing that organic combined with inorganic fertilization increased crop yield compared to chemical fertilization alone [23,33,34]. Li et al. [33] pointed out that organic fertilizers provided rich carbon and nitrogen to the soil. In addition, organic fertilization improves soil physicochemical properties, promoting root expansion, which in turn increases the absorption of water and nutrient in deeper soils, thereby increasing grain yield [23]. The CS treatment had a higher yield than the C treatment because of the abundant SOC (Table 1) and good soil structure (Table 3, Figs 1a and 1b). However, there was no significant difference between MM and MC treatments. This was related to MM treatment only significantly increasing SOC, compared to the MC treatment. The effect of organic fertilization on soil nutrients and structure was related to the experiment time. Previous studies have shown that when the experiment time is longer, crop yields under higher organic fertilization ratio treatments approach or even exceed those under lower organic fertilization ratio treatments [35]. For example, Dai et al. [13] showed that more than 70% organic fertilization combined with inorganic fertilization significantly improved crop yield compared with the lower organic fertilization proportion in the combined application of organic and inorganic fertilization for more than 30 years. With the increase in experiment time, a higher proportion of organic fertilization application (MM) in this study is expected to further improve crop (rapeseed and maize) yields.

Pearson correlations showed that TN, TK, AK, SBD, and WA<0.053 were the strongest correlates of both rapeseed and maize yield, followed by SOC, FC, WA>2 and WA0.250–2.000 (Fig 4). However, compared to the C treatment, long-term organic fertilization (MCS, MC, and MM) and straw addition (CS) treatments had no significant effect on TN and TK. This implies that long-term organic fertilization and straw addition promoted crop yield mainly by increasing AK, SOC, FC and WA>0.250, and reducing SBD and WA<0.053.

## 5 Conclusion

In this experiment, long-term organic fertilization (MCS, MC, and MM) significantly increased crop yields (rapeseed yield increased by 21.2~36.0%, and maize yield increased by 9.1~11.8%), soil organic carbon (SOC), alkali-hydrolyzed nitrogen (AN), available phosphorus (AP), available potassium (AK), soil porosity (SP), field capacity (FC), and the content of 0.250–2.000 mm large aggregates in soil water-stable aggregates (WA0.250–2.000), compared to chemical fertilizer (C); they also significantly decreased the soil bulk density (SBD) and WA<0.053. Similarly, straw addition (CS and MCS) treatments also had significant effects on soil nutrients, structure, and yield, compared to the C and MC treatments, respectively. However, compared to the MC treatment, applying more organic fertilizers (MM) had no significant effect on soil nutrients, structure, and yield. Compared to other fertilization regimes, MCS treatment has a greater capacity to increase soil nutrients, improve soil structure, and promote crop yields. This study provides a theoretical basis for reasonable fertilization in the rapeseed–maize rotation system in the southwestern arid areas of China.

## Supporting information

S1 File The supporting information file is provided in the form of data tables that support the findings reported in the paper. (XLSX)
